# Glutamic Acid Decarboxylase Injection Into Lymph Nodes: Beta Cell Function and Immune Responses in Recent Onset Type 1 Diabetes Patients

**DOI:** 10.3389/fimmu.2020.564921

**Published:** 2020-10-09

**Authors:** Rosaura Casas, Fabrícia Dietrich, Hugo Barcenilla, Beatriz Tavira, Jeanette Wahlberg, Peter Achenbach, Johnny Ludvigsson

**Affiliations:** ^1^ Division of Pediatrics, Department of Biomedical and Clinical Sciences, Faculty of Medicine and Health Sciences, Linköping University, Linköping, Sweden; ^2^ Department of Endocrinology, Region Östergötland, Linköping, Sweden; ^3^ Institute of Diabetes Research, Helmholtz Zentrum München, and Technical University of Munich, School of Medicine, Forschergruppe Diabetes, Munich, Germany; ^4^ Crown Princess Victoria Children’s Hospital, Region Östergötland, Linköping, Sweden

**Keywords:** autoantigen, immunotherapy, GAD-alum, type 1 diabetes, Vitamin D, intra-lymphatic treatment, lymph-node

## Abstract

In spite of intensive treatment Type 1 diabetes leads to serious complications. Preservation of residual beta cell function makes the disease milder, facilitates treatment, prevents complications and increase survival. So far immune interventions have had limited effect, and some serious adverse events and risks. In an open pilot trial we aimed to improve efficacy of GAD-alum treatment using lymph-node administration in combination with oral vitamin D. Here we report the clinical effect and focus on biomarkers for response to treatment. Patients (n = 12) aged 12 to 24 years with recent onset of Type 1 diabetes received 4 μg GAD-alum into lymph-node at day 30, 60, and 90, and oral Vitamin D 2000 U/d, days 1 to 120. Beta cell function was estimated by Mixed Meal Tolerance Tests. GADA, GADA subclasses, GAD_65_-induced cytokines and proliferation, and T cells markers were analyzed. The treatment was tolerable with no adverse events. Fasting C-peptide and insulin requirement remained stable at 15 months, while HbA1c was lower than baseline. Stimulated C-peptide showed no change at 6 months but declined after 15 months (81% of baseline). Eleven patients remained in partial remission (IDAAC < 9). Patients (n = 9) with better clinical outcome had reduced proportion of IgG1 and increased IgG2, IgG3, and IgG4, increased IL-10 secretion, and reduction of proliferation and CD8^+^ T cells activation. Patients with poorer clinical response had higher baseline levels of GAD_65-_induced cytokines and T-cell activation, and an increased ratio of effector/central memory T cells. Intra-lymphatic GAD treatment combined with Vitamin D might preserve beta cell function and improve clinical course in T1D. Patients with less benefit have a different quality of immune response both before and after treatment.

**Clinical Trial Registration:**

clinicaltrials.gov, identifier NCT02352974.

## Introduction

Subcutaneous administration of glutamic acid decarboxylase (GAD)_65_ formulated with aluminum hydroxide (GAD-alum) preserved residual insulin secretion in children and adolescents with recent-onset Type 1 diabetes (T1D) (1), but such treatment did not show efficacy in subsequent phase II (2) and phase III (3) trials. However, a meta-analyses suggested that the treatment most probably has beneficial effects (4). Heterogeneity of T1D makes it unlikely that a single agent or type of treatment will be effective in all patients. Hence, we need more knowledge to tailor treatment strategies and select suitable participants in T1D intervention trials. Small pilot studies can be useful in gaining insight before conducting large trials (5, 6).

In an attempt to render the presentation of GAD_65_ antigen more efficient, GAD-alum was injected into the lymph-nodes of six young adults with T1D in an open-label clinical trial. Vitamin D per os was added as a support (7). The treatment was safe, and preservation of C-peptide after 6 months appeared to be promising (8). Therefore, inclusion was increased, and also children included. This it to our knowledge the first clinical trial when autoantigen is given directly into lymph-nodes. Being a first-in-human pilot trial conclusions have to interpreted with caution. Here we give a preliminary report of the clinical effect in relation to immune response after 15 months.

## Materials and Methods

### Study Design and Participants

The GAD antigen into lymph-nodes (DIAGNODE-1) is a single center open-labeled pilot clinical trial. Patients aged 12 to 24 years diagnosed with T1D, according to American Diabetes Association criteria, with less than 180 days duration were screened. Twelve children and young adults (4 females, 8 males; 12·6–23·1 years old) (**Supplementary Figure 1**) were eligible if fasting C-peptide was ≥ 0·12 nmol/L (0·36 ng/ml) and GAD_65_ antibodies levels (GADA) > 63·2 U (detection limit at 95^th^ percentile), but <50 000 U. Full eligibility criteria are detailed in the study protocol. The protocol and consent documents were approved by appropriate independent ethics committees. All patients and caregivers of children gave their oral and written informed consent. The trial was approved by the Research Ethics Committee, Linköping University, Sweden (Dnr 2014/153-31) and by the Medical Product Agency, Uppsala, Sweden.

The Full Analysis Set (intention-to-treat) for the DIAGNODE-1 trial was defined as the population of patients who received at least one injection of GAD-alum (Diamyd^®^), taken part in the baseline visit and at least completed one follow-up visit. The control group was selected from the TN08 trial (2) evaluating Diamyd^®^ and placebo, with patients in the age range 12-24 receiving placebo, completed baseline, and at least one follow-up visit (**Supplementary Table 1**).

### Procedures

Each patient received a primary injection of 4 μg each of GAD-Alum (Diamyd Medical, Stockholm) into an inguinal lymph node administrated by help of ultrasound technique, followed by two booster injections with one-month interval. They also received Vitamin D (Calciferol) in oral solution (2000 U/d) for 4 months, starting 1 month prior to first GAD-alum injection. All patients received intensive diabetes management, following the Swedish Guidelines. They were evaluated at baseline, 6 months, and 15 months with clinical examination, blood samples, and a Mixed Meal Tolerance Test (MMTT) (9). As this was a pilot trial, there was no single specified primary endpoint, but the following parameters were prespecified:

- Change in fasting C-peptide and C-peptide (90 min value and AUC_mean__0-120_ min) during an MMTT from baseline to month 6 and 15 months.
- Change of glycated hemoglobin (HbA1c) from baseline.
- Change of exogenous insulin dose (per kg body weight and 24 h) from baseline.
- Immunomodulatory effect of the treatment, with special emphasis in GAD-induced T cell responses, cytokine secretion, and GADA subclass distribution.

### Laboratory Tests

Laboratory analyses were performed at Linköping University, Sweden. Blood and serum samples were collected at baseline and after 1, 2, 3, 6, and 15 months. Samples were drawn during the morning hours and peripheral blood mononuclear cells (PBMCs) were isolated within 24 h using Leucosep (Greiner Bio One) according to the manufacturer’s instructions.

Serum C-peptide was determined using a solid phase-two side enzyme immunoassay (Mercodia, Uppsala), and results were validated with the inclusion of a Diabetes Antigen Control Human (Low/High) (Mercodia, Uppsala, Sweden). Inter and intra assay variation were 7% and 4% respectively.

### Serum Antibodies and IgG Subclasses

Serum GAD autoantibodies (GADA) titers were estimated in duplicate by means of a radio-binding assay, using ^35^S-labeled recombinant human GAD_65_ (rhGAD_65_) as previously described (10).

GADA IgG 1, 2, 3, and 4 subclasses were measured by radio-binding assays (11) using IgG subclass specific biotin-labeled mouse-anti-human monoclonal antibodies bound on Streptavidin Sepharose High Performance beads (GE Healthcare Life Sciences, Freiburg, Germany) (12). Results were expressed as delta cpm (IgG subclass-specific cpm − anti-rat IgM cpm) and converted to arbitrary units (AUs) proportional to the GADA IgG subclass-specific delta cpm of a local standard serum.

### Lymphocyte Proliferation Assay

Proliferative responses were analyzed in PBMCs in the presence of 5 μg/ml rhGAD_65_ (Diamyd Medical, Stockholm, Sweden), CD3/CD28 beads (Gibco, Life Technologies AS, Oslo, Norway), or in medium alone. Stimulation index (SI) was calculated as the mean of triplicates in the presence of stimulus divided by the mean of triplicates with medium alone.

### Cytokine Secretion Assay

PBMCs were cultured for 7 days with 5 μg/ml rhGAD_65_ (Diamyd Medical, Stockholm, Sweden) or in medium (AIM-V with β-mercaptoethanol) at 37°C in 5% CO_2_, as previously described (13). The cytokines IL-2, IL-5, IL-10, IL-13, IL-17, tumor necrosis factor (TNF-α), and interferon (IFN-γ) were measured in cell supernatants using Bio-Plex Pro Cytokine Panel (Bio-Rad, Hercules, CA, USA). Data was collected using the Luminex 200™ (Luminex xMAP™ Corporation, Austin, TX USA). The antigen-induced secretion was calculated by subtracting the spontaneous secretion (i.e. secretion from PBMCs cultured in medium alone) from the one following stimulation with GAD_65_.

### Flow Cytometry

After washing in PBS containing 0·1% BSA, PBMCs were stained with Alexa-700-conjugated anti-CD3 (BD Biosciences Cat# 557943, RRID:AB_396952), Pacific Blue-conjugated anti-CD4 (BD Biosciences Cat# 558116, RRID:AB_397037), allophycocyanin (APC)-H7-conjugated anti-CD8 (BD Biosciences Cat# 560179, RRID:AB_1645481), PerCP-Cy5·5-conjugated anti-CD45RA (BD Biosciences Cat# 563429, RRID:AB_2738199), phycoerythrin (PE)-conjugated anti-CCR7 (BioLegend Cat# 353203, RRID:AB_10916391), FITC-conjugated anti-CD127 (Thermo Fisher Scientific Cat# 11-1278-42, RRID:AB_1907342), and PE-Cy7-conjugated anti-CD25 (Thermo Fisher Scientific Cat# A15857, RRID:AB_2534627). Then, cells were fixed and permeabilized using FOXP3 staining buffer set (Thermo Fisher Scientific Cat# 00-5523-00), according to the manufacturer’s instructions. Cells were then stained with APC-conjugated anti-FOXP3 (Thermo Fisher Scientific Cat# 17-4776-41, RRID:AB_1603281) and acquired on a FACS Aria III (BD Biosciences) running FACS Diva v8 software (Becton Dickinson). Data were analyzed using Kaluza v1·3 (Beckman Coulter).

### Statistical Analysis

Paired t test was applied to calculate fasting and stimulated C-peptide, glycated hemoglobin, and insulin dose differences within a group. For the evaluation of significant differences between groups, Welch’s t test was used. As the immunological data did not follow normal distribution, Wilcoxon test was applied to analyze differences within groups. For the calculation of significant differences between groups, Mann-Whitney test was used. Spearman’s rank coefficient was applied for correlations. A probability level of <0·05 was considered statistically significant. Calculations were performed using GraphPad Prism 8·0·1 for Windows (GraphPad Software, La Jolla, CA, USA).

## Results

### Clinical Responses at 15 Months

**Figure 1:**
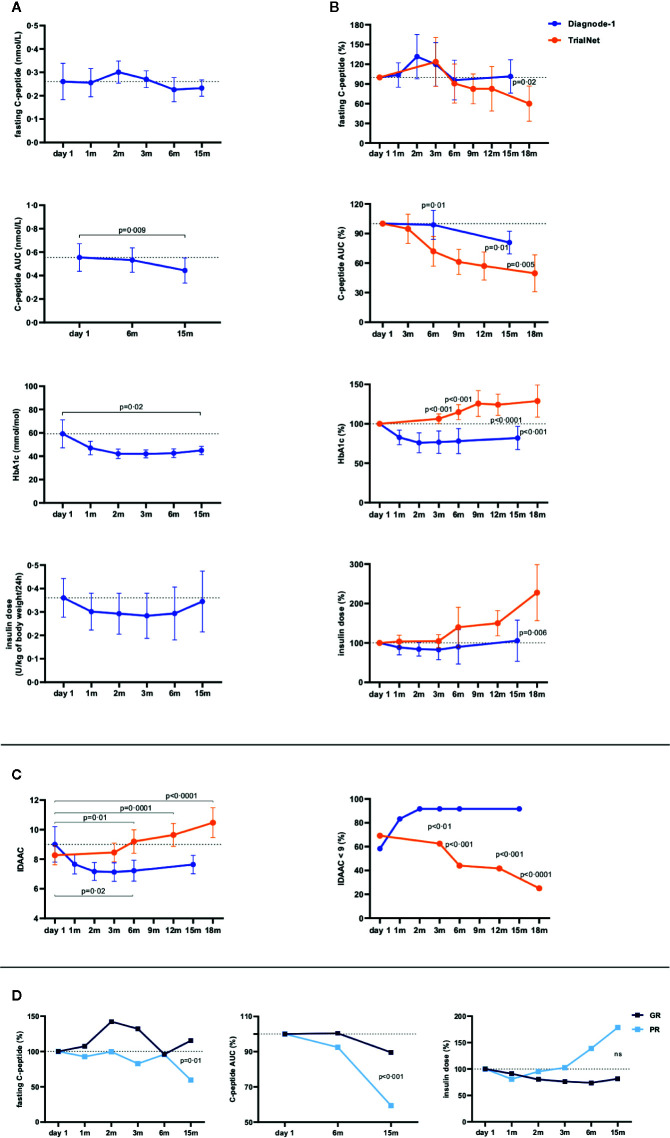
Clinical response from baseline (day 1) levels to 15 months. **(A)** Mean fasting and stimulated C-peptide (nmol/L), HbA1c (mmol/mol), and insulin dose (U/kg of body weight/24 h) in patients receiving GAD-alum injections into the lymph-node (n = 12) over time. **(B)** Patients receiving GAD-alum injections into the lymph-node (n = 12, blue circles) were compared to a group of patients with similar age who received placebo in another study where GAD-alum was given subcutaneously (n = 26, orange circles). Mean percentages of fasting and stimulated C-peptide, HbA1c, and insulin dose were compared between the groups at 3 and 6 months, and data from 15 months from the lymph-node group was compared to 12- and 18-months results from the placebo group. **(C)** Mean IDAAC in patients receiving GAD-alum injections into the lymph-node (n = 12, blue circles) and placebo (n = 26, orange circles) (left). Percentage of patients in partial remission, defined as IDAAC <9 (right). **(D)** Mean percentages of fasting and stimulated C-peptide (AUC), and insulin dose in patients stratified into Good Responders (GR, n = 9, loss < 30% AUC, dark blue squares) and Poor Responders (PR, n = 3, lowest quartile, loss ≥ 30% AUC, light blue squares) according to their C-peptide preservation at 15 months. Error bars indicate 95% CI. Differences between time points were determined by paired t Test. Welch’s t test was applied to calculate differences between groups.

The treatment was easy and tolerable, with no adverse events related to the treatment, except for mild transient reaction at the injection site in a few patients. Fasting C-peptide remained stable at 15 months. Stimulated C-peptide, measured as the area under the curve (AUC), showed no change at 6 months (mean AUC 98%), but declined after 15 months in relation to baseline (to 81%). There was no change in insulin dose from baseline to 15 months, while HbA1c decreased (**Figure 1A**). Fasting and stimulated C-peptide, HbA1c, and insulin dose was compared with data from patients who received placebo subcutaneously in a different GAD-alum trial (2). Some points for the samples differed between the studies, thus data at 15 months from the lymph-node group was compared to 12- and 18-months results from the control group. We observed less decline of fasting and stimulated C-peptide and lower insulin dose and HbA1c at 15 months in the DIAGNODE-1 patients compared to 12 and 18 months in controls (**Figure 1B**). Individual data are shown in **Supplementary Table 2**.

Definition of partial remission as IDAAC < 9 (14) showed that 7/12 (58%) DIAGNODE-1 patients had IDAAC <9 at baseline; 11/12 (91%) at 15 months. In contrast, partial remission decreased in the control group over time from 18/26 (69%) at baseline to 10/24 (41%), and 5/20 (25%) at 12 and 18 months respectively (**Figure 1C**).

Patients were then stratified according to their C-peptide preservation at 15 months into Good Responders (GR, n = 9, no loss of fasting C-peptide and loss of C-peptide AUC< 30%) and Poor Responders (PR, n = 3, decreasing fasting C-peptide and loss of C-peptide AUC ≥ 30%) (**Supplementary Table 2**). GR individuals had a better preservation of stimulated C-peptide (**Figure 1D**, **Supplementary Table 2**), and their fasting C-peptide remained stable throughout the study and was higher than in the PR at 15 months (**Figure 1D**). Insulin requirement at 15 months was 19% lower in the GR than at baseline while insulin demand increased by 79% in PR patients (**Figure 1D**).

### Immune Responses at 15 Months

**Figure 2:**
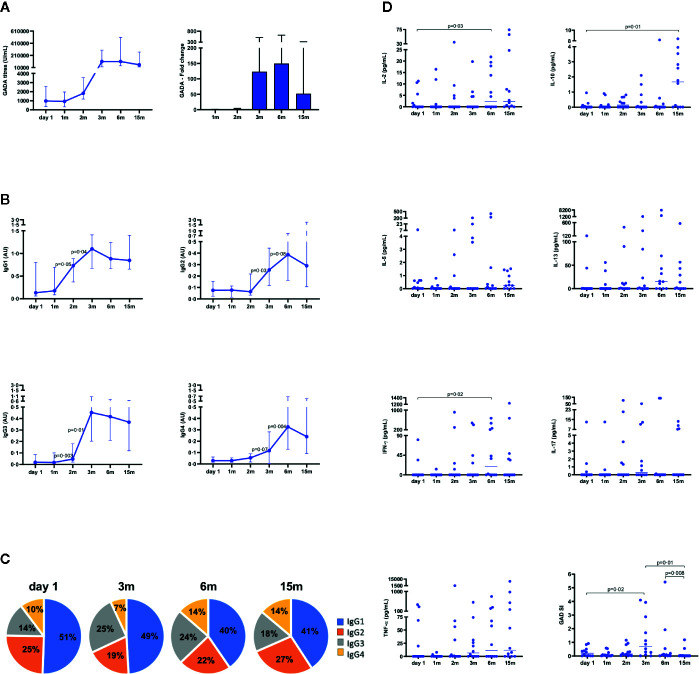
Immune response from baseline (day 1) to 15 months in patients (n = 12) who received GAD-alum injections into the lymph-node. **(A)** Median values and fold change of GADA titers (U/ml). **(B)** Median levels of IgG1, IgG2, IgG3, and IgG4 GADA subclasses are shown as arbitrary units (AUs). Error bars indicate interquartile range. **(C)** GADA IgG subclass relative distribution at baseline (day 1), 3, 6, and 15 months. Frequencies of each subclasses were calculated with respect to the combined sum of the AUs of the four subclasses in each sample. **(D)** Cytokine secretion and proliferation induced by GAD_65_ upon *in vitro* PMBCs stimulation. Levels of IL-2, IL-5, IL-10, IL-13, IL-17, IFN-γ, and TNF-α cytokines detected by Luminex in PBMCs supernatants after 7 days culture in medium alone or in the presence of GAD_65_ (5 µg/ml). GAD_65_-induced cytokine secretion levels are given after subtraction of spontaneous secretion from each individual and expressed as pg/ml. Proliferative responses to GAD_65_ in PBMCs cultured for 3 days with GAD_65_ (5 µg/ml), CD3/CD28 beads, or medium, and thereafter cells were pulsed with [^3^H] thymidine and harvested. Proliferation is expressed as stimulation index (SI) and calculated from the mean of triplicates in the presence of stimulus divided by the mean of triplicates with medium alone. Horizontal lines represent the median. Differences within the same group were calculated using Wilcoxon paired test.

A 52-fold change of GADA levels was observed at 15 months compared to baseline titers (**Figure 2A**). Analysis of the longitudinal changes of the GADA IgG 1-4 subclasses showed that IgG1 and IgG3 increased after the first injection, and were further enhanced after the second injection with no changes after the third dose, while IgG2 and IgG4 increased after the second injection, and were boosted by the third dose (**Figure 2B**).

Distribution of IgG subclasses, calculated as frequency of each subclass with respect to the combined sum of the AUs of all subclasses in each sample, showed a reduced proportion of IgG1 and a marked increase of IgG2, IgG3, and IgG4 at 15 months (**Figure 2C**), in line with previous results at 6 months (12).

The two first injections of GAD-alum induced secretion of IL-17 and TNF-α. The third dose additionally enhanced IL-2, IL-5, IL-13, IFN-γ, while IL-10 was first detected at 15 months and significantly increased in relation to baseline levels (**Figure 2D**). GAD_65_-induced proliferation was significantly enhanced by the second injection, but the third dose reduced T-cell proliferation, that was undetectable in almost all patients at 15 months (**Figure 2D**).

**Figure 3:**
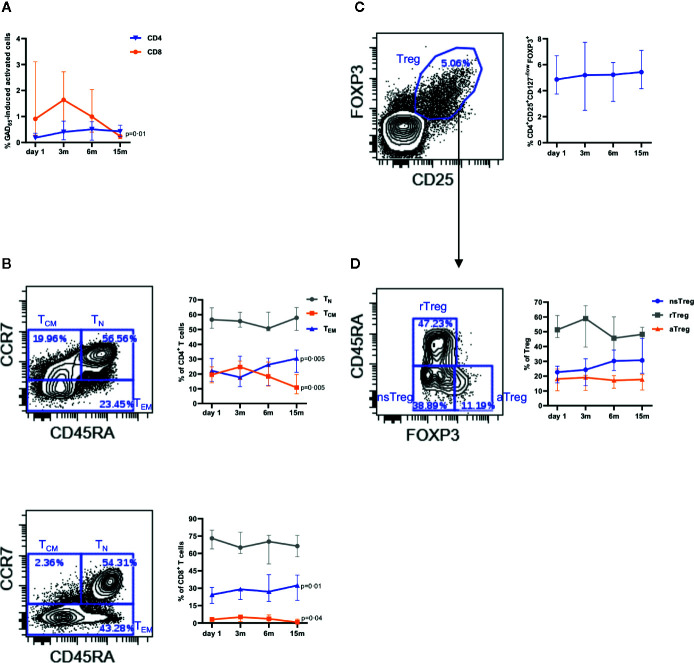
Effect of intra-lymphatic injection of GAD-alum (n = 12) on T cells and cells with regulatory phenotype. **(A)** Median percentage of activated (CD25^+^CD127^+^) CD4^+^ (blue triangles) and CD8^+^ (orange circles) T cells after stimulation with GAD_65_ (5 µg/ml). **(B)** Gating strategy for naïve (T_N_, CD45RA^+^CCR7^+^, gray circles), central memory (T_CM_, CD45RA^-^CCR7^+^, orange squares), and effector memory (T_EM_, CD45RA^-^CCR7^-^ and CD45RA^+^CCR7^-^, blue triangles) cells, and median percentage within CD4^+^ (up) and CD8^+^ (down) T cells. **(C)** Representative dot plot showing the expression of FOXP3 and CD25 on CD4^+^ T cells and median percentage of regulatory T cells within CD4^+^ T cells. **(D)** Gating strategy and median percentages for non-suppressive (nsTreg, CD45RA^-^FOXP3^low^, blue circles), resting (rTreg, CD45RA^+^FOXP3^low^, gray squares), and activated (aTreg, CD45RA^-^FOXP3^high^, orange triangles) regulatory T cells. Bars indicate interquartile range. Differences between time-points within the same group were calculated using Wilcoxon paired test.

The percentages of GAD_65_-induced CD4^+^ and CD8^+^ T cells did not vary along the study. Addition of the activation markers CD25 and CD127 showed that GAD_65-_stimulation mainly induced activated CD8^+^ T cells in baseline samples (**Figure 3A**), and that the proportion of these cells decreased significantly at 15 months (**Figure 3A**). T cell differentiation was determined according to expression level of CD45RA and CCR7 as näive (T_N_, CD45RA^+^CCR7^+^), central memory (T_CM_, CD45RA^−^CCR7^+^), and effector memory (T_EM_, CD45RA^−^ CCR7^-^ and CD45RA^+^CCR7^-^) cells. Higher proportion of T_EM_ than T_CM_, both on CD4^+^ and CD8^+^ T cells, was observed at baseline (ratio EM/CM 1·4 and 24·5, respectively, **Figure 3B**). We found a reduction of the T_CM_ fraction from baseline to 15 months in parallel to an increase of T_EM_ (ratio EM/CM 3·26 and 32·5) (**Figure 3B**).

The percentage of cells with Treg phenotype (CD4^+^FOXP3^+^CD25^high^CD127^low/−^) in non-stimulated PBMCs was slightly modified, but not significantly increased (5% to 6%, **Figure 3C**). Further analysis of Treg based on the expression of FOXP3 and CD45RA did not reveal variations in the activated effector pool (FOXP3^high^ CD45RA^−^). However, a progressive increment of the non-suppressive cells (FOXP3^low^CD45RA^−^) and a reduction of the resting (FOXP3^low+^CD45RA^+^) fraction, but not statistically significant, was observed (**Figure 3D**). Stimulation of samples with GAD_65_ did not show changes in any of the Treg subpopulations.

### Biomarkers of Clinical Outcome

**Figure 4:**
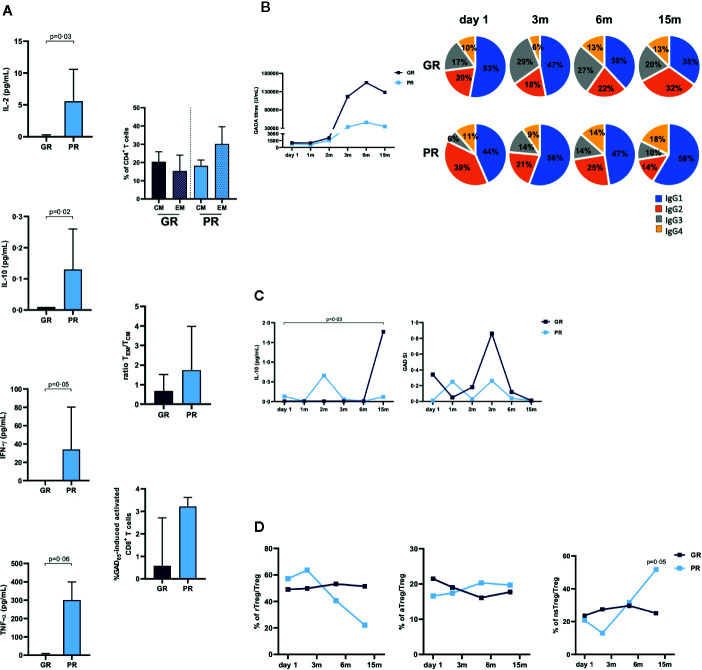
Immune responses in relation to clinical outcome. Patients were stratified into Good Responders (GR, n = 9, loss < 30% AUC, dark blue bars or squares) and Poor Responders (PR, n = 3, lowest quartile, loss ≥ 30% AUC, light blue bars or squares) according to their C-peptide preservation at 15 months. **(A)** Median baseline levels of GAD_65_-induced IL-2, IL-10, IFN-γ, and TNF-α cytokines detected by Luminex in PBMCs supernatants after 7 days culture in presence of medium or GAD_65_ (5 µg/ml). GAD_65_-induced cytokine secretion levels are given after subtraction of spontaneous secretion from each individual (pg/ml). Percentages of CD4^+^central memory (T_CM_, CD45RA^-^CCR7^+^) and effector memory (T_EM_, CD45RA^-^CCR7^-^ and CD45RA^+^CCR7^-^) T cells, ratio T_EM_/T_CM_, and GAD_65_-induced activated CD8^+^ T cells. Bars indicate interquartile range. **(B)** Median values of GADA titers and GADA IgG subclass relative distribution. Frequencies of each subclasses were calculated with respect to the combined sum of the AUs of the four subclasses in each sample. **(C)** Median levels of GAD_65_-induced IL-10 cytokine and PBMCs proliferation. **(D)** Median percentages of resting Treg (rTreg), activated Treg (aTreg), and non-suppressive Treg (nsTreg) within total Treg. Statistical differences between groups were calculated using Mann-Whitney test, differences between time-points within the same group were calculated with Wilcoxon paired test.

Stratification of the patients into GR and PR showed that baseline levels of GAD_65_-induced IL-2, IL-10, IFN-γ, and TNF-α were higher in PR patients (**Figure 4A**). GAD_65_-stimulation also induced CD4^+^ T cells with higher T_EM/_T_CM_ ratio in PR (1·73) than in GR (0·68) patients. Furthermore, activation of CD4^+^ and CD8^+^ T cells seemed more pronounced in PR samples (mean % of activated cells, CD4^+^: 0·17 vs 0·46 and CD8^+^: 1·33 vs 2·37) (**Figure 4A**).

Following the treatment, GADA subclasses distribution was characterized by a marked reduction of IgG1 in the GR patients, while IgG1 dominated the IgG subclasses proportion in PR subjects (**Figure 4B**). Higher levels of GAD_65_-induced IL-10 were observed in the GR patients, while IL-10 was undetectable at 15 months in PR patients (**Figure 4C**). Changes in the immune response at 15 months also showed a significant increase of non-suppressive FOXP3^low^CD45RA^−^ Tregs in the PR samples (**Figure 4D**).

## Discussion

Our study suggests that GAD-alum administration directly into lymph-nodes of T1D patients in combination with oral vitamin D may result in better preservation of C-peptide than seen in T1D patients of similar age (2, 15). Vitamin D is supposed to improve efficacy by its effect on the immune system, and the design of this study makes it impossible to discriminate the effect of GAD-alum from the effect of Vitamin D. However, in other studies with the same Vitamin D treatment, we have only seen some support for using Vitamin D, but no clear effect on beta cell preservation (16). The idea of a main effect due to GAD-alum is supported by the results showing that main changes after treatment are all antigen-specific, while non-specific immunomodulatory effects were not observed. It has been also described that Vitamin D alone in the dose administrated under the study does not give as pronounced effect on the immune system (7)

In contrast to some types of immune interventions, this treatment was easy and tolerable for the patients with no adverse events related to the treatment except for mild transient reaction at the injection site in a few patients. Reduction of proliferation and enhanced IL-10 suggest the induction of antigen-specific regulatory responses and tolerance as part of the immunological effect. Our data indicate a different quality of the immune response to GAD_65_ in patients with less benefit, both pre and post treatment. The immune response induced by lymph-node injections of GAD-alum seems to differ in many aspects from that when higher doses were injected subcutaneously (10, 13, 17). Recall response to GAD_65_ at 15 months showed reduction of CD8^+^ T cells activation, as well as a decrease of the T_CM_ fraction, and increase of T_EM_ cells both in CD4^+^ and CD8^+^ T cells. Although immunological memory is displayed by both fractions, T_CM_ have limited effector function while T_EM_ can rapidly produce effector cytokines upon antigenic stimulation (18). The apparent predominant proportion of CD4^+^ T_EM_ cells at baseline was due to higher T_EM_ fraction in the poor responders. This was an interesting observation, as CD4^+^ T_CM_ are the predominant memory cells in the blood compartment (18). Higher GAD_65_-induced cytokine secretion was also found in baseline samples from the poor responders. When generated, antigen-specific memory T cells have to compete with pre-existing cells for access to survival factors (19). Thus, it might be possible that the frequency of pre-existing specific-effector T cell and a pro-inflammatory environment when autoantigens are administered, determine the nature of memory T cells generated by the treatment. If so, our data support the idea that selective depletion of specific-memory effector cells preceding autoantigen administration might improve efficacy.

Several cytokines were induced as part of immune response to GAD_65_. Among them, IL-5 and IL-13, which together with IL-4, are the major effector Th-2 cytokines, known to stimulate the switch of antibody isotypes in B cells, and T-helper cells differentiation, protect tissues from ongoing damage, and has potent anti-inflammatory activities, both *in vitro* and *in vivo* (20–22). Notably, rapid enhancement of GADA correlated with IL-5, and it was characterized by a shift in the subclass’s distribution, with a reduction of IgG1 following the third GAD-alum injection. Secretion of IL-5 started to increase at 3 months, when IgG1 levels began to wane, while the levels of the other subclasses continued rising. It can be argued that secretion of IFN-γ and TNF-α Th1-associated cytokines might not be desirable. However, cytokines can exert different effects depending on their concentrations and microenvironment (23–25). A role for TNF-α in regulating Th2-type responses as a critical component of IL-13-mediated protective effect (26) has been attributed to its Th2-promoting activity and influenced by the cytokine milieu (21). Thus, under the right circumstances, cytokines can exhibit either Th1 or Th2-promoting activities.

One of the postulated effects of antigen immune therapy is the induction of antigen-specific Tregs. The slight increase of Tregs observed at 15 months was observed when Tregs were defined by the expression of FOXP3, CD25^high^, and lacking CD127, commonly used for Tregs definition (27). However, further dissection of the Tregs pool revealed that the apparent increase of Tregs was explained by the increment of the percentage of non-suppressive cells, while the population of activated more suppressive Tregs remained unaltered. It cannot be excluded that the scarce number of GAD_65_-specific cells precluded their identification.

Modifications following therapy included the loss of GAD65-induced proliferation in parallel to an increase of GADA titers. This observation is in line with a previous study where increased antibody titers and lower proliferation against insulin has been shown in a prevention trial using intranasal insulin given to at-risk individuals, suggesting induction of tolerance (28). Reduction of proliferation together with enhanced levels of IL-10 and reduction of GAD65-induced activation of CD8+ T cells might suggest that induction of tolerance was part of the immunological effect. Thus, our data suggest the induction of antigen-specific regulatory responses and tolerance as part of the immunological effect of autoantigen administration into the lymph-nodes.

The increasing consensus on the heterogeneity of T1D brings focus to the matter that, as in many other autoimmune diseases, many of the patients participating in clinical trials have benefit from the treatments, while others have not (1, 29–31). Indeed, results from trials considered not effective in T1D are similar to those observed in other diseases, suggesting that the treatments work as effective in T1D (32). In this first-in-human pilot trial a large number of patients was not allowed. Consequently, it lacks power to show statistically significant results. We are aware of that the natural course in T1D means that some patients may have residual beta cell function for rather long time even without any intervention (33) and get a rather long partial remission defined as IDAAC <9 (34). This might even be prolonged in patients who participate in clinical trials, being extra motivated to treat their disease in an active way. Nevertheless, comparison of the results with data from placebo patients from another trial showed that those seemed to have a more rapid decline of C-peptide and less good clinical course than patients in our study. We are aware of that the controls are historical, but participating in a rather recent randomized, double-blind, placebo-controlled trial, using GAD-alum in the actively treated arm, with patients using similar modern therapy and similar follow-up. Still, conclusions need to be cautious.

In an effort to find biomarkers for clinical response we divided the patients into so-called Good Responders (9 patients) and Poor Responders (3 patients), based on the response criteria used in other studies (29). We found an interesting difference in immune response between Good Responders and Poor Responders but are aware of the very low number of patients. Although our results should be interpreted with caution, we report them to stimulate other studies to confirm or disapprove.

Intra-nodal administration of an autoantigen was easy to perform and tolerable for the patients. Although this open-label pilot study was not designed to measure efficacy, it looks as if the decline in secreted C-peptide slowed down, and the daily insulin requirement and HbA1c decreased in the patients. Here we also show that the pre-existing antigen-specific immune responses may be important for the outcome, raising the question whether further immunological parameters than positivity to autoantibodies should be used for patient selection. Subjects considered Good Responders showed immunological changes upon *ex vivo* stimulation including production of IL-10, lack of proliferation, reduction of CD8^+^ cells activation, and switch of GADA subclasses. Our results require confirmation in a well-powered randomized double-blind placebo-controlled trial (which is ongoing). It might be interesting to use intra-nodal administration of other autoantigens, or even together with other immunotherapeutic agents, both in T1D and other autoimmune diseases.

## Data Availability Statement 

The raw data supporting the conclusions of this article will be made available by the authors, without undue reservation.

## Ethics Statement

The studies involving human participants were reviewed and approved by Research Ethics Committee, Linköping University, Sweden (Dnr 2014/153-31). Written informed consent to participate in this study was provided by the participants’ legal guardian/next of kin.

## Author Contributions

FD, BT, and HB performed experiments and analyzed data. FD and HB contributed to prepare the manuscript. RC conceived the study, designed data set and data analysis, and wrote the first draft of the manuscript. JL had the idea, designed DIAGNODE, conceived the study, and reviewed the manuscript. JW recruited and followed patients. PA performed the analysis of GADA subclasses. JL and RC are guarantors of this work and, as such, had full access to all the data in the study and take responsibility for the integrity of the data, and the accuracy of the data analysis. All authors contributed to the article and approved the submitted version.

## Funding

This work was supported by Barndiabetesfonden (Swedish Child Diabetes Foundation), Swedish Diabetes research foundation, and an unrestricted grant from Diamyd Medical. The funders were not involved in the study design, collection, analysis, interpretation of data, the writing of this article or the decision to submit it for publication.

## Conflict of Interest

The authors declare that the research was conducted in the absence of any commercial or financial relationships that could be construed as a potential conflict of interest.

## Abbreviations

GADA, glutamic acid decarboxylase autoantibodies; GAD-alum, glutamic acid decarboxylase formulated in aluminum hydroxide; IDAAC, insulin dose adjusted HbA1c; LN, lymph node; PBMC, peripheral blood mononuclear cells; SC, subcutaneous; Th cell, T helper cell; T1D, type 1 diabetes.
