# Supplementary material for: Glutamic Acid Decarboxylase Injection Into Lymph Nodes: Beta Cell Function and Immune Responses in Recent Onset Type 1 Diabetes Patients
Source: Front Immunol. 2020 Oct 9;11:564921. doi: 10.3389/fimmu.2020.564921 (PMC7583358; doi:10.3389/fimmu.2020.564921)
Supplement: Supplementary file 1 [file Table_1.docx]

***Supplementary material***

**Supplementary Figure**


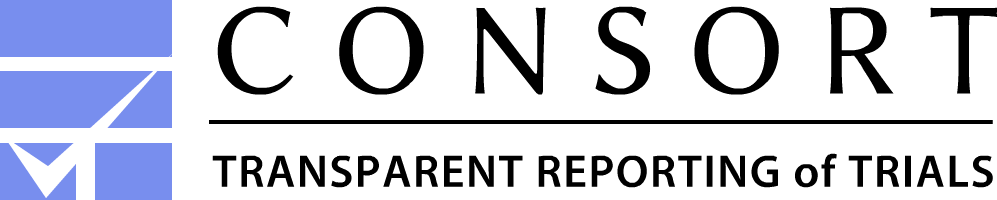


**CONSORT 2010 Flow Diagram**

Excluded (n=8)

  Did not meet inclusion criteria (n=6)

  Declined to participate (n=2)

Analysed (n=12)

Lost to 450 days follow-up (n=0)

Allocated to intervention (n=12)

## Allocation

## Analysis

## Follow-Up

Randomized (n=12)

## Enrollment

Assessed for eligibility (n=20)

**Supplementary Figure 1.** Flow of patients in DIAGNODE-1, a pilot study of the effect of intra-lymphatic GAD-alum in recent onset Type 1 diabetes.

**Supplementary Table**

**Supplementary Table 1.** Baseline data comparing the DIAGNODE-trial patients with the control group (treated with placebo in another GAD-alum trial (1)).

| **Variable** | **DIAGNODE-1 (n=12)** | **Controls (n=26)** |
| --- | --- | --- |
| **Gender distribution, n (%)** |  |  |
| **Female** | 4 (33) | 8 (31) |
| **Male** | 8 (67) | 18 (69) |
|  |  |  |
| **Age, mean (SD), y** | 19·08 (4·44) | 16·65 (3·52) |
|  |  |  |
| **Fasting C-peptide, mean (SD), nmol/L** | 0·26 (0·12) | 0·39 (0·21) |
|  |  |  |
| **C-peptide AUC, mean (SD), nmol/L** | 0·55 (0·19) | 0·80 (0·35) |
|  |  |  |
| **HbA1c, mean (SD), mmol/mol** | 59·17 (18·89) | 47·55 (13·13) |
|  |  |  |
| **Insulin dose, mean (SD), U/kg /24 h** | 0·36 (0·13) | 0·44 (0·24) |
|  |  |  |
| **IDAAC, mean (SD)** | 9·00 (1·89) | 8·37 (1·63) |
|  |  |  |
| **IDAAC <9 (%)** | 7 (58) | 18 (69) |

Abbreviations: AUC, area under the curve; HbA1c, glycated hemoglobin; IDAAC, insulin dose adjusted HbA1c.

Reference

1. Wherrett DK, Bundy B, Becker DJ, DiMeglio LA, Gitelman SE, Goland R, et al. Antigen-based therapy with glutamic acid decarboxylase (GAD) vaccine in patients with recent-onset type 1 diabetes: a randomised double-blind trial. Lancet (2011) 378:319–27. doi: 10.1016/S0140-6736(11)60895-7

**Supplementary Table 2.** Baseline (day 1) and 15 months clinical parameters in each patient who received GAD-alum injections into the lymph-nodes.

|  |  |  |  | **day 1** | | | | **15 months** | | | | **Δ variable change (%)^*^** | | | |  |
| --- | --- | --- | --- | --- | --- | --- | --- | --- | --- | --- | --- | --- | --- | --- | --- | --- |
| **Patients** |  | **Gender** | **Age (years)** | **Fast C-pep** | **C-pep AUC** | **Insulin dose** | **HbA1c** | **Fast C-pep** | **C-pep AUC** | **Insulin dose** | **HbA1c** | **Fast C-pep** | **C-pep AUC** | **Insulin dose** | **HbA1c** | **Responders** |
| 1 |  | F | 22 | 0·17 | 0·36 | 0·46 | 52 | 0·24 | 0·39 | 0·54 | 41 | 41 | 8 | 16 | -21 | GOOD |
| 2 |  | M | 22 | 0·26 | 0·85 | 0·25 | 58 | 0·26 | 0·68 | 0·28 | 54 | 0 | -20 | 10 | -7 |  |
| 3 |  | M | 23 | 0·12 | 0·43 | 0·29 | 66 | 0·22 | 0·35 | 0·15 | 52 | 83 | -18 | -48 | -21 |  |
| 4 |  | F | 21 | 0·25 | 0·74 | 0·45 | 68 | 0·28 | 0·81 | 0·11 | 41 | 12 | 8 | -76 | -40 |  |
| 5 |  | M | 22 | 0·26 | 0·42 | 0·29 | 103 | 0·18 | 0·36 | 0·07 | 45 | -31 | -14 | -77 | -56 |  |
| 6 |  | M | 23 | 0·16 | 0·38 | 0·55 | 78 | 0·21 | 0·27 | 0·34 | 44 | 31 | -28 | -39 | -44 |  |
| 7 |  | F | 23 | 0·24 | 0·54 | 0·22 | 41 | 0·28 | 0·41 | 0·08 | 37 | 17 | -25 | -66 | -10 |  |
| 8 |  | M | 12 | 0·23 | 0·52 | 0·36 | 37 | 0·26 | 0·54 | 0·41 | 46 | 13 | 5 | 13 | 24 |  |
| 9 |  | M | 13 | 0·25 | 0·52 | 0·29 | 66 | 0·18 | 0·40 | 0·58 | 40 | -28 | -23 | 100 | -39 |  |
| 10 |  | M | 20 | 0·21 | 0·36 | 0·47 | 54 | 0·12 | 0·22 | 0·54 | 51 | -43 | -38 | 15 | -6 | POOR |
| 11 |  | F | 15 | 0·58 | 0·85 | 0·15 | 37 | 0·24 | 0·53 | 0·46 | 39 | -59 | -38 | 210 | 5 |  |
| 12 |  | M | 13 | 0·40 | 0·69 | 0·53 | 50 | 0·32 | 0·38 | 0·59 | 49 | -20 | -46 | 11 | -2 |  |

Abbreviations: F, female; M, male; Fast C-pep, Fasting C-peptide (nmol/L); C-pep AUC, C-peptide area under the curve (nmol/L); Insulin dose, U/kg of body weight/24 hours; HbA1c, glycated hemoglobin (mmol/mol).

^*^Percentage of change (%) of C-peptide, Insulin dose, and HbA1c from baseline (Δ variable change) were calculated.
